# Psycho-Neuroendocrinology in the Rehabilitation Field: Focus on the Complex Interplay between Stress and Pain

**DOI:** 10.3390/medicina60020285

**Published:** 2024-02-07

**Authors:** Mirjam Bonanno, Davide Papa, Antonio Cerasa, Maria Grazia Maggio, Rocco Salvatore Calabrò

**Affiliations:** 1 IRCCS Centro Neurolesi Bonino-Pulejo, 98124 Messina, Italy; mirjam.bonanno@irccsme.it (M.B.); roccos.calabro@irccsme.it (R.S.C.); 2 International College of Osteopathic Medicine, 20092 Cinisello Balsamo, Italy; davide.papa.dp99@gmail.com; 3 S’Anna Institute, 88900 Crotone, Italy; antonio.cerasa@irib.cnr.it; 4 Institute for Biomedical Research and Innovation (IRIB), National Research Council of Italy (CNR), 98164 Messina, Italy; 5 Translational Pharmacology, Department of Pharmacy, Health and Nutritional Sciences, University of Calabria, 87036 Rende, Italy

**Keywords:** stress, pain, neurorehabilitation, immune system, hypothalamus–hypophysis axis, pain matrix

## Abstract

Chronic stress and chronic pain share neuro-anatomical, endocrinological, and biological features. However, stress prepares the body for challenging situations or mitigates tissue damage, while pain is an unpleasant sensation due to nociceptive receptor stimulation. When pain is chronic, it might lead to an allostatic overload in the body and brain due to the chronic dysregulation of the physiological systems that are normally involved in adapting to environmental challenges. Managing stress and chronic pain (CP) in neurorehabilitation presents a significant challenge for healthcare professionals and researchers, as there is no definitive and effective solution for these issues. Patients suffering from neurological disorders often complain of CP, which significantly reduces their quality of life. The aim of this narrative review is to examine the correlation between stress and pain and their potential negative impact on the rehabilitation process. Moreover, we described the most relevant interventions used to manage stress and pain in the neurological population. In conclusion, this review sheds light on the connection between chronic stress and chronic pain and their impact on the neurorehabilitation pathway. Our results emphasize the need for tailored rehabilitation protocols to effectively manage pain, improve treatment adherence, and ensure comprehensive patient care.

## 1. Introduction

Stress occurs due to physical or psychological stimuli that disrupt the organism’s homeostasis. These stimuli are defined as stressors, and the physiological and behavioral changes that occur after exposure to stressors constitute the stress response [1]. A complex interplay mediates the stress response, involving the nervous, endocrine, and immune mechanisms, resulting in the activation of the sympathetic–adrenomedullary (SAM) axis, the hypothalamic–pituitary–adrenal (HPA) axis, and the immune system [2,3]. The stress response prepares the body for challenging situations or mitigates tissue damage after a trauma [3,4]. However, persistent exposure to a stressor (repetitive and prolonged) favors maladaptive reactions, leading to depression, anxiety, cognitive impairment as well as worsening neurological and cardiovascular diseases [5,6]. From a biological perspective, stress is defined as the activation of the HPA axis, which results in the release of adrenal steroids triggered by the secretion of adrenocorticotropic hormone (ACTH) from the pituitary gland [7]. The release of corticotropin-releasing factor (CRF) by the hypothalamus controls ACTH stimulation in response to various stressors [2,3,7]. Furthermore, the activation of HPA promotes the release of glucocorticoids into the systemic circulation. Consequently, glucocorticoids play important roles in response to stress. Any dysregulation of these delicate systems can lead to various stress-related complications. For example, glucocorticoids affect cognitive functions, causing short-term and reversible deficits in episodic and spatial memory after stressful events [8,9].

Moreover, stress appears to be associated with pain, according to some theories. Pain is specifically defined by the International Association for the Study of Pain (IASP) as “an unpleasant sensory and emotional experience associated with actual or potential tissue damage or described in terms of such damage” [10,11]. Researchers have adopted two mutually non-exclusive models linking pain and stress. The first model considers pain as a form of stress that places a burden on the organism. In particular, chronic pain (CP) leads to “wear-and-tear”, also known as allostatic overload, in the body and brain due to the chronic dysregulation (i.e., over-activity or inactivity) of physiological systems that are normally involved in adapting to environmental challenges [12]. These wear-and-tear changes can compromise human well-being, affecting social and occupational life [12]. People who experience persistent pain (i.e., chronic pain) may exhibit deficits in decision-making. Moreover, the fear of movement due to CP, i.e., inactivity, could lead to a more sedentary lifestyle, increasing the allostatic overload [13]. In the second model, patients experience unforeseeable stress, such as migraine episodes, which triggers a vicious cycle of “feed-forward” maladaptive physiological responses, including inflammation and brain damage, leading to increased vulnerability to persistent pain [14]. These two theories draw from stress literature to provide either a causal explanation for the onset and persistence of chronic pain or its long-term consequences, without necessarily contradicting one another [15]. Additionally, they emphasize that discomfort and stress can form a vicious cycle of unhelpful reactions to environmental difficulties, which can harm one’s wellbeing. 

Managing CP in neurorehabilitation presents a significant challenge for healthcare professionals and researchers, as there is no definitive and effective solution for it [16]. Patients suffering from neurological disorders (ND) often complain of CP, which significantly reduces their quality of life (QoL) [16]. In this context, non-invasive and non-pharmacological therapies have been developed to address CP resulting from various causes [17]. These therapies can be categorized into physical therapies, psychological interventions, and complementary and alternative approaches [17,18]. 

The main objective of this narrative review is to examine the correlation between stress and pain and their potential negative impact on the rehabilitation process. Our secondary objective is to describe the most relevant interventions used to manage stress and pain in the neurological population. 

## 2. Effect of Stress on Endocrine and Immunological Systems and CP

The impact of stress on the endocrine and immunological systems is a dynamic process characterized by non-specific changes in the organism when exposed to stressors [19]. Stress is a subjective response influenced by individual cognitive processing, enabling more adaptive physiological and behavioral responses to navigate stressful situations [3,4,5,6]. Contrary to common perception, stress is not inherently negative. Instead, it enables individuals to react appropriately to internal or environmental stimuli, showcasing the unique ability of humans to process stressful situations based on personal experiences [3,4,5,6]. To understand the changes in organisms during stress, it is insightful to consider the “fight or flight” response in animals facing danger. This response involves various systems, including the musculoskeletal, respiratory, and cardiovascular systems, all coordinated by the hypothalamus, as highlighted by Cannon [20,21]. Stressors of various origins activate different chemical mediators (cytokines, neurotransmitters, neuropeptides, etc.), which in turn trigger the paraventricular nuclei of the hypothalamus (NPV) and initiate two distinct stress pathways [15]. The neural stress pathway involves the stimulation of the locus coeruleus, which enhances alertness and activates the sympathetic autonomic nervous system, preparing the body for fight or flight. The locus coeruleus releases norepinephrine, which optimizes brain reactivity, information processing, concentration, and cognitive vigilance [21]. Simultaneously, the sympathetic system activates visceral organs, promoting a general state of readiness. This stress pathway culminates in the adrenal medulla, which releases catecholamines (adrenaline, norepinephrine, and dopamine), amplifying the effect of the sympathetic nervous system [22]. The chemical stress pathway, mediated by the HPA axis, initiates a cascade that results in the production of cortisol from the adrenal cortex [23]. The HPA axis plays a crucial role in coordinating the body’s stress response by releasing classic stress hormones such as ACTH and cortisol. Cortisol, with immediate effects following the neural response, mobilizes energy resources, increases glucose availability, and supports catecholamine action on the cardiovascular system [20,21,22,23]. 

### 2.1. Introduction to Stress Signaling Pathways

The body’s response to stress is a complex orchestration of intricate signaling pathways that ensure a swift and adaptive reaction to external challenges. Two primary pathways, i.e., the nervous stress pathway and the chemical pathway of stress, play pivotal roles in this dynamic process [24].

The nervous stress pathway involves the sympatho-adreno-medullary system, wherein information from the paraventricular nuclei of the hypothalamus directly reaches the locus coeruleus [25]. This activation enhances brain reactivity, alertness, and cognitive vigilance, while the orthosympathetic system readies the body for immediate action. The release of norepinephrine and the subsequent activation of the adrenal medulla reinforce the effects of the sympathetic nervous system, creating a coordinated response known as the “fight or flight” reaction [21,25].

Conversely, the chemical pathway of stress operates through the HPA axis. Triggered by a chemical signal via the pituitary gland and adrenal cortex, this pathway results in the production of cortisol [26]. The HPA axis regulates various physiological processes, including metabolism and immune function. Cortisol, with its rapid mobilizing effect on energy resources, complements the immediate neural response, ensuring a comprehensive adaptation to stressors [25,26].

Stress significantly influences the perception of pain by impacting various neural pathways, particularly those involving mesolimbic–cortical dopamine neurons. The nucleus accumbens, a crucial component of the mesolimbic dopaminergic pathway, emerges as a key player in the modulation of pain and exhibits distinct responses to stressful events [27]. Moreover, the intricate relationship between chronic pain and these stress pathways adds another layer to this complexity. Chronic pain conditions contribute to dysregulations in cytokine production, further influencing both the nervous and chemical stress pathways. As we explore each pathway in detail, we unravel the profound impact of stress and pain on the body’s endocrine, immune, and nervous systems [28].

In the subsequent sections, we delve into the specifics of each pathway, exploring their nuanced roles in the broader context of stress responses, with a particular focus on the intricate interplay between stress, chronic pain, and immune function.

### 2.2. The Nervous Stress Pathway: Sympatho-Adreno-Medullary 

From the paraventricular nuclei of the hypothalamus, information directly reaches the locus coeruleus, activating the entire brain (increased vigilance), and the medullary nuclei of the orthosympathetic system [21,29]. The locus coeruleus is the main center of norepinephrine release. The release of norepinephrine is lowest during sleep, increases during wakefulness, and reaches much higher levels when new unexpected stimuli arrive and in situations of stress or perceived danger. The function of the locus coeruleus is to increase brain reactivity and the speed of information processing of the sensory and motor pathways, making them more efficient, stimulating concentration, attention, and cognitive vigilance, and generating a state of mental acuity, determination, and physical and cognitive speed [21,22,23,24,25,26,27,28,29]. The orthosympathetic system activates a series of visceral organs (vessels, heart, lungs, glands) and inhibits others (stomach, bladder, intestine), thus putting the organism in a state of general activation suitable for fight or flight [30]. An efferent pathway therefore originates which, bypassing the synapses, directly reaches the adrenal medulla, triggering the release of catecholamines (adrenaline, norepinephrine, and dopamine, reinforcing the effect of the sympathetic nervous system (stress nervous pathway) [31]. Thus, the locus coeruleus essentially mobilizes the brain for action, while the sympathetic system mobilizes the body [21]. 

### 2.3. Chemical Pathway of Stress: Hypothalamic-Pituitary-Adrenal Axis 

Following a chemical signal, via the pituitary gland and the adrenal cortex, the HPA’s activation ultimately results in the production of cortisol. In this sense, this axis plays a critical role in orchestrating the body’s response to stress, initiating a cascade of hormonal events aimed at adapting to and coping with difficult situations [32]. This hormonal response is crucial for regulating various physiological processes, including metabolism and immune function [33]. In the short term, cortisol has a general mobilizing effect of the body’s energy resources necessary to support the fight/flight reaction. This hormonal response follows the nervous response, which is immediate (about 20 min later). Cortisol has notable physiological, metabolic and hepatic effects, increasing the energy substrates (glucose) available to allow the body to cope with the increased energy demands linked to stress conditions and supporting the action of catecholamines on the cardiovascular system [34].

Furthermore, the relationship between CP and cytokines adds another layer to the intricate interaction between stress and the endocrine and immune systems. Studies [35,36] suggest that CP conditions may contribute to the dysregulation in cytokine production. Elevated levels of pro-inflammatory cytokines, such as IL-1, IL-6, and TNF, are often observed in individuals with CP. This surge in cytokines, influenced by the prolonged stress response, not only amplifies the inflammatory environment but establishes bidirectional communication with the HPA axis [37]. The dysregulated cytokine profile may further influence the HPA axis, potentially contributing to a feedback loop that exacerbates both chronic pain and stress responses, further showing the broad impact of stress on immune function and overall homeostasis [38].

The production of catecholamines by the adrenal medulla is increased by cortisol and in a short timeframe it stimulates the immune system. Finally, it increases vigilance. When in excess, it generates states of anxiety and fear and induces passive behaviors of submission and avoidance [39]. The release of cortisol and catecholamines into circulation targets the immune system. During physiological stress, the immune system releases pro-inflammatory cytokines (IL-1, IL-6, TNF), which reach the brain through humoral and nervous pathways [40]. This neurobiological network reactivates if the inflammatory response proves inadequate, prompting the hypothalamus to release CRH and other chemical mediators once again [19,40]. The hypothalamic–pituitary–adrenal axis is self-regulating (negative feedback), as the hypothalamus and pituitary glands control the circulating levels of cortisol via specific receptors, thus allowing the activation or inhibition of cortisol levels [41]. The duration and nature of stressors contribute to the variability of HPA axis responses. In fact, in a condition of chronic stress, especially in subjects in which the response to stress or in any case the ability to adapt to stressful situations is not very effective, the self-regulation mechanism begins to no longer function correctly [42]. Overstimulation is caused by the various chemical mediators listed above. At the hypothalamic and pituitary level, there will instead be a reduction in the receptors, leading to incorrect feedback of the signals arriving from the periphery and alterations in adequately dealing with any kind of stressor, either cognitive or chemical–physical [43]. In summary, stress has a profound impact on the endocrine and immunological systems through the complex modulation of the HPA axis. The multiple responses of the HPA axis, which include classical stress hormones and additional regulatory molecules, highlight its central role in maintaining homeostasis during stress. 

### 2.4. Physiological Effects of Chronic Pain of Endocrine System 

The basic physiological effect of pain on the endocrine system is one of severe stress. When pain signals reach the brain, the hypothalamus is activated in the same way as with chronic stress, and three hormones, i.e., corticotropin-releasing hormone, gonadal-releasing hormone, and thyroid-releasing hormone, are released [44]. In turn, these hormones promote the release of ACTH, follicle-stimulating hormone, luteinizing hormone, and thyroid stimulating hormone into the serum from the anterior pituitary gland [45]. The target organs of these hormones are the adrenal, gonad, and thyroid glands, which release into the serum the hormones necessary for pain control, including cortisol, pregnenolone, DHEA, testosterone, progesterone, estrogen, triiodothyronine (T3), and thyroxine (T4) [44]. When severe pain occurs, serum hormone levels are increased. If pain persists over time, the hormonal system becomes unable to tolerate chronic stress due to pain, and hormonal serum levels decrease below normal. Not coincidentally, serum levels of these molecules can be used as biomarkers to determine pain severity. 

### 2.5. Navigating the Impact of Stress and Pain on the Immune System

The relationship between stress, pain, and the immune system unfolds with immediate and prolonged effects. Originating from evolutionary responses to environmental threats, stress triggers physiological reactions that intricately shape the human immune system. Over three decades of research, more than 300 studies have elucidated the dynamic interplay between psychological stress, pain, and immune responses [45]. 

Within the immune system’s natural and specific components, stress and pain induce notable alterations. Natural immunity, orchestrated by granulocytes and natural killer cells, provides swift, generalized defense, while specific immunity, governed by lymphocytes, offers precise responses [46]. Immune assays, scrutinizing cells, proteins, or functions, reveal the intricate nature of stress-induced immune changes [45,46].

The pathways connecting stress, pain, and the immune system involve sympathetic fibers, hormonal axes, and behavioral factors. Evolving models acknowledge the nuanced effects of acute and chronic stressors [25,26]. The biphasic model posits that acute stress enhances immune responses, while chronic stress suppresses immune responses. Cytokine shift models reconcile stress-related disease outcomes by considering the simultaneous enhancement and suppression of immune components [47].

The immune system’s vulnerability to stress and pain-induced changes is influenced by factors like age and existing health conditions. While healthy individuals may exhibit flexibility in responding to stress, aging and diseases compromise this adaptability, potentially leading to stress-related immune dysregulation with clinical implications [48,49]. 

The impact of stress and pain on the immune system is manifested in various symptoms. Acute stress may enhance immune responses, fostering adaptability in the face of immediate challenges. In contrast, CP often leads to immune suppression, potentially increasing susceptibility to infections and disease. CP, in particular, emerges as a crucial factor influencing the immune response. Prolonged pain conditions may lead to immune suppression, potentially increasing susceptibility to infections and other health complications [45]. This nuanced understanding of the short-term and enduring effects of stress and chronic pain on the immune system provides valuable insights for holistic health considerations.

## 3. Learning Mechanisms for Pain and Stress

The experience of pain and stress involves intricate cognitive and behavioral processes, drawing upon various learning mechanisms that shape our responses to these stimuli. Classical and operant conditioning, habituation, sensitization, and observational learning intricately weave together to influence how individuals perceive, express, and adapt to pain and stress. Understanding these learning mechanisms unveils the complex dynamics that contribute to the chronicity and intensification of these experiences [50]. In this exploration, we will delve into each learning mechanism, unraveling their roles in the modulation of pain and stress responses and examining how these processes interconnect to form the intricate tapestry of our behavioral and physiological reactions. 

### 3.1. Classical Conditioning (Pavlovian Learning)

Classical conditioning involves the association of a neutral stimulus with a biologically potent stimulus, leading to a learned response [51]. In the context of pain, this process contributes to the development of anticipatory reactions to cues associated with pain. For instance, stimuli that were neutral initially become linked with painful experiences, creating a conditioned response that can be triggered independently of the actual pain stimulus. The neural pathways established through classical conditioning contribute to the emotional and physiological aspects of pain perception.

### 3.2. Operant Conditioning

Operant conditioning focuses on behavior modification through reinforcement or punishment. In the context of chronic pain, behaviors such as adopting specific postures or expressing pain verbally can be influenced by external factors [52]. Positive reinforcement, such as attention, sympathy, or relief, may strengthen these painful behaviors, contributing to their persistence. Conversely, a lack of reinforcement for healthy behaviors or activities can impede the development of adaptive coping mechanisms. Operant conditioning thus plays a crucial role in shaping the ongoing cycle of pain experiences and responses.

### 3.3. Habituation and Sensitization

Beyond classical and operant conditioning, habituation and sensitization contribute significantly to the modulation of pain and stress responses [53]. Habituation occurs when a stimulus is repeatedly presented without a significant outcome, leading to a decreased response over time. An everyday example is adapting to background noise, like living near a train station. Initially perceived as annoying, habituation results in a reduced awareness of the stimulus. 

Conversely, sensitization involves an increased response to repeated exposure to the same stimulus. In the context of chronic pain, this heightened sensitivity can manifest as increased pain perception (hyperalgesia) and the experience of pain from typically non-painful stimuli (allodynia). Stressful situations can exacerbate sensitization, amplifying the overall impact on pain experiences [54].

### 3.4. Observational Learning

Observational learning, as proposed by Albert Bandura, emphasizes the acquisition of behaviors by watching others [55]. Children, for example, learn pain-related behaviors by observing their parents or significant adults. If a parent exhibits avoidance behaviors or expresses distress due to pain, the child may adopt similar responses [56]. This observational learning extends beyond the family unit, as individuals may adjust their pain-related behaviors based on the responses of those around them. Moreover, observational learning plays a crucial role in the social transmission of pain behaviors. Empathy for pain activates similar brain areas as the direct experience of pain, indicating that witnessing pain in others can elicit physiological and emotional responses. This interconnectedness underscores the influence of social dynamics on the perception and expression of pain and stress [57].

## 4. Revealing the Pain Matrix and Central Sensitization Phenomena 

Pain pathways, also known as the “pain matrix” or “neuromatrix”, comprise a complex network of cortical areas (e.g., prefrontal cortex, amygdala, nucleus accumbens, anterior cingulate cortex) that become active to protect an individual from noxious stimuli as well as somatosensory stimulation [58,59]. The pain matrix or neuromatrix theory of pain proposes that pain is a multidimensional experience produced by characteristic “neurosignature” patterns of nerve impulses generated by a widely distributed neural network, the “body–self neuromatrix”, in the brain [60]. In a chronic stress situation, the major corticolimbic areas, modulating stress response, change their pattern of connectivity leading to a morphological and permanent shift into the stressed neuromatrix. Then, it determines an altered behavior per se, but also an adequate control of stress response [61]. 

In addition, noxious stimuli are transmitted through nociceptors by primary afferent Aδ and C fibers, which have cell bodies located in the dorsal root ganglion and synapse with neurons in the spinal dorsal horn [62]. During signal transduction, various neurotransmitters are released, including glutamate, calcitonin gene-related peptide (CGRP), and substance P. From the dorsal horn, projection neurons decussate at the ventral commissure and ascend in the lateral spinothalamic tract to the ventral posterolateral nuclei of the thalamus [62,63]. Successively, information is transmitted to the somatosensory cortex and periaqueductal grey matter (PAG) [64]. However, other brain areas are involved in the transmission of nociceptive information, including the amygdala, hypothalamus, and nucleus accumbens through the spino-reticular and spino-mesencephalic tracts [65]. Moreover, descending pain modulatory systems involve the PAG and rostral ventral medulla (RVM). This latter serves as the primary output node in the descending modulation of nociception [66]. It receives an input from the PAG, and sorts diffuse bilateral projections to the dorsal horn, arriving at multiple levels. When a peripheral injury occurs, the interaction between neuronal and glial cells influences the development of pain hypersensitivity [64,65,66]. Glial cells, such as microglia, astrocytes, oligodendrocytes, and radial cells, have supportive and protective functions in both the central and peripheral nervous systems [67]. Neurotransmitters released by glial cells involved in pain pathways regulate pain processing at the spinal and supraspinal levels. Moreover, glial cells release inflammatory chemokines and cytokines that facilitate pain transmission by coupling to neuronal glutamate receptors [64,67]. When nociceptive stimuli are prolonged over time due to injury or inflammation, long-term alterations and plastic changes occur in the central nervous system (CNS) [68]. This phenomenon is called “central sensitization” (CS). CS is associated with heightened responses in primary afferent fibers, as well as elevated spontaneous activity and excitability of dorsal horn neurons and their receptive field regions [69]. Finally, the CS mechanism involves the intervention by various neuromodulators, which can have both inhibitory and excitatory effects, depending on the specific molecule and the receptor it binds. For example, glutamate signaling through postsynaptic N-methyl-D-aspartate (NMDA) receptors. When NMDA receptors are activated, ion channels open, allowing calcium to influx. Calcium flux is crucial for synaptic plasticity in both excitatory and inhibitory synapses. Synaptic plasticity has the potential to sensitize the central nociceptive system, leading to pain hypersensitivity and chronic pain [69,70]. In this context, the presence of hyperalgesia can also occur, due to the release of neurotrophic factors like brain derived neurotrophic factor (BDNF), which is involved in synaptic plasticity and is involved in CS. In addition, gamma aminobutyric acid (GABA) can contribute to CS if its inhibitory tone is decreased, causing an imbalance between excitation and inhibition [71]. On the other hand, the presence of opioid peptides can reduce the release of glutamate and substance P, which are involved in CS. In detail, opioid receptors are abundantly disseminated in both primary afferent neurons and dendrites of post-synaptic neurons. Their presence can lead to a reduction in perception of pain and an increase in pain tolerance. In addition, endocannabinoids, including anandamide and 2-arachidonoylglycerol, act on cannabinoid receptors (CB1 and CB2) and play a role in pain modulation. The release of endocannabinoids has a fundamental role in suppressing excitatory neurotransmission and attenuating CS [72]. 

Traditionally, the prevailing belief held that the induction of CS by noxious stimuli necessitated a prolonged, intense, and repetitive application of the stimulus. However, contemporary insights challenge this notion, revealing that sustained peripheral nociceptive input may not be imperative for the initiation of CS. This is because alterations in the characteristics of neurons within the central nervous system, seemingly detached from peripheral input, can precipitate CS. The resultant outcome is an augmentation of pain sensitivity, achieved by modifying the sensory responses elicited by routine inputs, encompassing subthreshold innocuous tactile stimulation. This is the reason why patients affected by neurological disorders can feel pain without damage to the peripheral tissues [73]. Another phenomenon that can be observed in neurological disorders is the “cross organ sensitization”, a phenomenon where pain in one organ can lead to pain in another organ that is distant from its origin [74]. For example, patients with a migraine and/or fibromyalgia can experience CS as well as cross-organ sensitization. However, there is no direct correlation between these two phenomena.

## 5. Psychological Effects of Chronic Pain and Stress-Related Pain Perception 

Psychological effects related to CP are linked to the involvement of various brain structures, such as the primary somatosensory cortex, secondary somatosensory cortex, anterior cingulate cortex, prefrontal cortex, insular cortex, amygdala, thalamus, and cerebellum, which are associated with the perception of pain [75,76]. Several research studies [77,78,79] have examined the influence of CP on emotions and psychological well-being. For example, Baliki et al. [79] found that chronic musculoskeletal pain is associated with increased activity of the prefrontal cortex (PFC) and nucleus accumbens. These brain areas are also involved in emotion, motivation, and reward-related behaviors [75,76,79]. In this sense, emotional and motivational triggers can influence the intensity and degree of pain experienced. 

In addition, CP is associated with maladaptive neuroplastic changes in the grey matter, particularly in the corticolimbic structures and emotional cerebral pathways, which are involved in processing pain. Several neuroimaging studies [75,76,77,78,79,80] have demonstrated that chronic painful experiences are associated with a decrease in grey matter density in the insular cortex, primary somatosensory cortex, motor cortex, hippocampus, amygdala, and NAc. Given the functions of these brain regions, it is clear that CP can easily contribute to emotional and cognitive problems. Furthermore, these maladaptive changes in plasticity may also occur in sensory conduction pathways from the peripheral nervous system to the central nervous system, contributing to the onset, progression, and persistence of CP [81,82]. 

CP shares similar neurobiological mechanisms with depression [83]. Research has shown that both chronic pain and depression can lead to a decrease in monoamine neurotransmitters, including serotonin, dopamine, and norepinephrine [84]. Specifically, dopamine, which plays an essential role in the forebrain, appears to be less responsive in the limbic pathways of individuals with CP. In particular, the dopamine receptor D2 is a protein involved in the onset of depression. Not surprisingly, individuals who experience chronic pain often also suffer from depression. This could be explained by the activation of similar brain areas in both cases. In fact, the brain regions involved in pain pathways, such as the prefrontal cortex, hippocampus, and amygdala, are similar to those involved in mood disorders [85,86,87]. Similarly, other psychological effects related to pain can occur, such as anxiety, anhedonia, sleep disturbances, and suicidal thoughts. It appears that individuals with chronic pain conditions are more likely to consider suicide than those without such conditions [88].

## 6. Chronic Stress and Pain and Its Implications in Neurorehabilitation 

Every year, people suffering from neurological disorders (e.g., stroke, traumatic brain injury, spinal cord injury, and neurodegenerative disorders) need to be submitted to specific neurorehabilitation pathway [89]. When neurological damage occurs, both motor and cognitive deficits are treated, although the presence of pain can limit recovery. The presence of pain in neurological disorders is very frequent and can be related to spasticity, musculoskeletal abnormalities/lesions, headache, and mixed pain [90]. Nonetheless, pain in patients who undergo neurorehabilitation is underreported and/or undetected. Indeed, there are no standard guidelines for the evaluation and management of pain for patients with neurological disorders as of yet [91]. One of the most important issues is that pain is negatively correlated with the recovery process, because patients experiencing pain are more likely to miss treatment sessions and/or be unable to participate to the full extent during such sessions [92]. This aspect can also have consequences for patients’ relatives and/or caregivers. In addition, patients affected by a brain injury—both vascular and traumatic—can manifest psychological symptoms like depression, which can worsen the perception of pain [18]. In summary, pain coupled with the presence of depression can worsen the recovery process and the adherence to the treatment. According to a recent systematic review and meta-analysis, multidisciplinary pain management programs may be a valid strategy for those patients suffering from severe or long-lasting pain [93]. 

### 6.1. Non-Pharmacological Management of Chronic Stress and Pain 

The non-pharmacological management of chronic stress and pain encompasses a variety of approaches, including both physical and psychological modalities [94]. 

### 6.2. Physical Approaches 

Among physical therapies, physical exercise is a powerful strategy to manage pain. Physical exercise not only reduces pain perception but also has a role in improving mood and reducing stress and depression, which are often associated with CP conditions. At the brain level, the rostral ventromedial medulla (RVM) plays a key role in exercise-induced analgesia, as well as the PAG and dorsal horn. Furthermore, exercise increases the circulating levels of endocannabinoids. After both aerobic and resistance exercise tasks, there is an increased expression of cannabinoid receptors in the brain, including the PAG, in healthy uninjured animals, but also humans [95]. 

TENS is particularly used for pain relief. It consists of the application of low-intensity electrical stimulation in the target body area. The underlying neurophysiological mechanism of functioning is based on the gate-control theory. At the peripheral level, TENS activate large, myelinated fibers Aβ which inhibit nociceptive Aδ fibers. In this way, the presynaptic inhibition of nociceptive stimuli occurs at the dorsal horn [96,97]. 

Acupuncture consists of the stimulation of traditional acupoints along meridians, according to traditional Chinese medicine. This approach is used to modulate pain, increasing the release of adenosine, which is an endogenous anti-nociceptive molecule. Neuroimaging studies with functional magnetic resonance revealed that acupuncture can modulate limbic areas involved in affective pain, also activating anti-nociceptive brain areas like PAG [98,99]. 

Yoga and tai chi are mind–body practices that may relieve both chronic pain and stress. Both practices combine physical exercise, balance and meditation. According to EEG studies, these two practices have an impact on theta, alfa, and gamma oscillations, which are associated with the sensory, cognitive and affective processing of pain. In addition, it seems that yoga and tai chi can promote the release of endogenous opioids, such as serotonin and dopamine, from the PAG, amygdala and nucleus accumbens (NAc) [100,101,102]. 

Manual therapy is referred to as a set of hands-on techniques [103]. Among these, osteopathic manipulative treatment (OMT) is focused on the global health of the subjects, acting in many body areas, even those distant from the pain localization. The tenet of OMT is based on the ability of the body to self-heal, and the osteopathic physician improves this process. Specifically, OMT includes the soft tissue, visceral techniques, resisted isometric muscle energy stretches, spinal manipulation, and cranial sacral therapy. It has been demonstrated that therapeutic touch induces a sort of relaxation associated with a decrease in stress markers, such as cortisol. At the brain level, OMT activates the ACC and AIC, both involved in pain processing. In addition, AIC has a role in the release of oxytocin, a hormone with powerful analgesic effects [104,105,106]. 

### 6.3. Psychological Approaches 

Cognitive behavioral therapy (CBT) is a type of psychotherapy with the purpose of orientating thoughts and behavior related to CP and stress. Through different techniques, like relaxation training, emotional regulation, and exposure techniques, CBT enhances positive thinking. At the biological level, CBT has a role in increasing endogenous opioids and decreasing inflammatory biomarkers like TNF-a and IL-6. In addition, CBT may induce changes in brain connectivity, influencing brain areas related to pain (PFC, ACC, amygdala, and insula). It regulates pain through “gating” mechanism, engaging descending modulatory pathways [107,108]. CBT holds the potential to influence hormonal regulation through its impact on psychological and emotional well-being. This therapeutic approach, centered on modifying patterns of thinking and behavior, extends its effects to physiological processes. CBT has demonstrated efficacy in stress management, a key factor in the hormonal balance [107]. By helping individuals to cope with stressors and reframe maladaptive thought patterns, CBT may contribute to a more balanced regulation of stress hormones, such as cortisol. The treatment of mood disorders, like depression and anxiety, is a common application of CBT. These conditions often involve imbalances in neurotransmitters like serotonin, dopamine, and norepinephrine. CBT’s effectiveness in improving mood may indirectly influence the equilibrium of these hormones. Sleep quality, closely tied to hormonal patterns like melatonin, can be positively impacted by CBT. Specifically, cognitive-behavioral therapy for insomnia has shown success in enhancing sleep duration and quality, demonstrating its influence on associated hormonal processes. CBT encourages positive lifestyle changes, targeting behaviors like diet and exercise. These modifications can have downstream effects on hormones related to metabolism and overall well-being. Within the realm of psychoneuroimmunology, CBT’s influence on psychological states may contribute to changes in the immune system, intricately connected with hormonal regulation. Enhanced mental well-being through CBT could positively impact both the immune and endocrine systems [107,108]. 

Mindfulness-based interventions aim to pay attention to bodily sensations, emotions, and thoughts to address both pain and stress. These techniques can act on cognitive, affective, and sensory facets of pain. Some studies have demonstrated that mindfulness interventions modulate brain areas involved in pain processing, like the prefrontal cortex, ACC, insula, thalamus, and amygdala. It seems that this kind of intervention may engage top-down regulation from the prefrontal cortex and ACC to inhibit maladaptive pain signaling. Like OMT, mindfulness techniques regulate the sympathetic–parasympathetic balance, normalizing the HPA and the cardiovascular and immune systems [109,110]. 

Virtual reality (VR) in computer-generated environments can reproduce reality with different degrees of immersion (e.g., non-, semi-, immersive VR). VR provides audio-visual feedback, promoting multi-sensorial stimulation. These multi-modal effects can induce neuroplastic changes by altering sensory perception and modulating pain-related neural processing. At the cellular level, VR also has a role in inhibiting molecular mechanisms involved in CS [111,112]. 

Non-invasive brain stimulation (NIBS) is an innovative rehabilitation treatment which is used to modulate cortical excitability, in order to promote functional recovery by boosting neuroplastic processes, enhancing the reorganization of neuronal “networks” of brain areas. Generally, two different types are distinguished: transcranial magnetic stimulation (TMS), with the repetitive TMS (rTMS) or intermittent θ-burst stimulation (i-TBS) types, and transcranial electrical stimulation (tES) with its different applications (tDCS, transcranial direct current stimulation; tACS, transcranial alternating current stimulation; tRNS, transcranial random noise stimulation). Some studies have shown that NIBS induces modifications of neurotransmitters, such as glutamate, GABA, serotonin and dopamine, which are critical for pain processing. Also, it has been demonstrated that NIBS promote changes in the “pain matrix”, which includes the thalamus, ACC, somatosensory cortex, and insula [113,114]. 

## 7. Discussion

**Figure 1:**
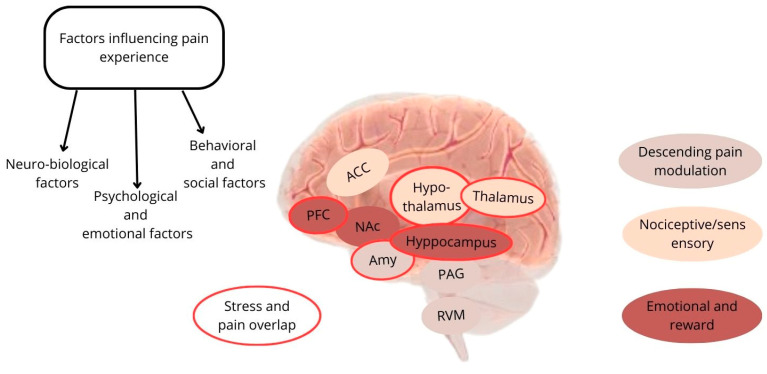
Schematic representation of the main factors and brain areas involved in pain and stress overlap. Legend: PFC, prefrontal cortex; ACC, anterior cingulate cortex; NAc, nucleus accumbens; Amy, amygdala); PAG, periaqueductal grey matter; RVM, rostral ventromedial medulla.

This narrative review primarily discusses the link between chronic stress and CP, emphasizing their negative impact on the rehabilitation process, especially in patients with neurological disorders. On the other hand, we identified the most commonly used rehabilitation strategies for managing pain and stress in this patient population. Chronic stress and CP can be viewed as being interconnected, sharing similarities in neuroanatomical and endocrinological pathways [12] (see Figure 1). 

However, these two aspects are less commonly investigated in the neurological population. It is known that psychological trauma and stressful life experiences can be considered key etiological factors of functional neurological disorders [115]. Stressful experiences can contribute to the risk of autoimmune diseases such as multiple sclerosis and myasthenia gravis through the immune system. On the other hand, stress can exacerbate symptoms of neurological diseases, such as epilepsy and progressive neurodegenerative diseases [116]. In addition, stress worsens the perception of fatigue, a debilitating symptom that is common in various neurological disorders. Finally, neurological symptoms can themselves be a source of stress, leading to high levels of distress [117]. Stress is commonly associated with the presence of pain, especially in people suffering from neurological diseases, although the management of chronic pain in this patient population is still a neglected issue [115,116,117]. In particular, rehabilitation goals mainly focus on the recovery of motor and cognitive functions, while the presence of pain is often underestimated and/or attributed to depression. This factor significantly contributes to reduced adherence to rehabilitation treatment, thus negatively affecting the outcomes. According to a systematic review [118], pain during exercise is a common barrier to adherence, highlighting the importance of strategies to alleviate pain. This is why clinicians need to gain a clear understanding of the patient’s pain experience and beliefs about pain, encouraging patients to pursue the rehabilitation path. There are various non-pharmacological interventions that can be used to alleviate pain [94]. In most cases, the appropriate use of OMT, VR, or NIBS and/or other physical and psychological strategies could be key factors in managing this issue. Even though these interventions are different, they share common mechanisms that produce analgesic effects. For example, most of the approaches discussed can modulate nociceptive signaling, influencing psychological and emotional aspects of stress and pain and limiting central sensitization underlying CP, while also promoting neuroplasticity in some cases. However, the heterogeneity of CP conditions and the individual response to treatment highlights the need to tailor rehabilitation protocols, considering the role of CP [94].

Future research in this field should prioritize the promotion of integrative and combination treatments. As outlined in the neuromatrix or pain matrix theory by Melzack [60], a multimodal approach that integrates physical, psychological, and complementary therapies could effectively target pain mechanisms at the peripheral, spinal, and supraspinal levels [119,120]. Nowadays, a variety of non-pharmacological treatments can be used to address CP and its numerous manifestations. This aspect can have a positive impact because patients can benefit from a variety of solutions. On the other hand, it can also be a limitation for clinicians when selecting the most suitable approach due to the multitude of treatments available. Emerging therapies like OMT are focused on a patient-centered perspective, and OMT seems to be safe and effective in the management of CP due to muscle-skeletal diseases [121,122]. It has been hypothesized that manipulative techniques can produce changes in the localized structure and function, helping to normalize neural sensory input and/or influence the reflex pathway and its activity in cortical and subcortical areas. In this sense, future studies should investigate the role of OMT in modulating the activation of the pain matrix in patients affected by neurological disorders suffering from CP. 

Moreover, novel computer science approaches such as machine learning and computational modelling are gaining popularity and have been proposed as promising innovative treatments for CP. However, the application of machine learning in pain research may be limited by the availability and quality of data. It relies on the maintenance of knowledge bases or the successful enrolment of many subjects in clinical studies [123]. In this context, there is another concern related to assessing pain. In fact, pain is a “subjective” perception, and measuring the serum levels of stress-related hormones may not be sufficient to assess pain severity. To achieve this goal, new research is needed in the field of omics sciences to discover novel and useful biomarkers. According to some authors [124], damage-associated molecular patterns (DAMPs) could provide a future solution for objectively assessing pain. In fact, DAMPs are molecules released upon cellular stress or tissue injury. They are regarded as endogenous danger signals because they induce potent inflammatory responses by activating the innate immune system during non-infectious inflammation [124]. In this vein, omics approaches provide a useful insight into the complexity of pain pathophysiology in humans. Diatchenko et al. [125] suggested that omics analysis is useful to further understand the association with a specific immune cell type or a gene, the overall pain mechanism, and the molecules involved in the resolution process. Despite these promising potential applications, we are still far away from their implementation in clinical practice, especially in the field of neurorehabilitation.

In the end, CP mechanisms should be further studied, even more so in the neurological population since this is a neglected issue. Translating these concepts into clinical approaches is imperative in order to administer safe and effective therapies, reducing costs related to hospitalizations and ensuring a better quality of life. Therefore, for a precision pain medicine approach, a multidisciplinary team is needed to examine specific patient’s needs.

## 8. Conclusions

This narrative review sheds some light related to the connection between chronic stress and chronic pain and their impact on the path of neurorehabilitation. It is evident that these elements are primarily studied from neuroanatomical, biological, and endocrinological perspectives. There are several unanswered questions related to pain in patients undergoing neurorehabilitation. These questions concern the prevalence of pain, the types of pain experienced, the need for specific treatment, the impact of pain on the neurorehabilitation outcome, and the quality of life after discharge. Despite these concerns, clinicians should meet patients’ needs and encourage them to pursue the recovery process. In this context, specific rehabilitation protocols are needed to manage pain effectively, promote treatment adherence, and take a comprehensive approach to patient care. Patients should be encouraged by clinicians to begin the recovery process without fear or anxiety about pain, as these feelings can significantly limit adherence to the treatment. Lastly, there is an urgent need for innovative methods to objectively assess pain. It can also be beneficial to monitor patients over time during the recovery process.

## Data Availability

Data sharing is not applicable.
